# Omental Cyst With Torsion: A Rare Culprit of Pediatric Acute Abdomen

**DOI:** 10.7759/cureus.50606

**Published:** 2023-12-15

**Authors:** Reem Albejais, Ghaiah Alharbi, Eman Al-Ali, Nuwayyir Aldawsari, Tawfeeq Johar

**Affiliations:** 1 General Practice, King Faisal University, Hofuf, SAU; 2 General Surgery, Dallah Hospital, Riyadh, SAU

**Keywords:** computed tomography, laparotomy, acute abdomen, omental torsion, omental cyst

## Abstract

Acute abdominal pain in pediatric patients poses a diagnostic challenge due to diverse etiologies, ranging from benign to life-threatening conditions. Omental cysts, though rare, constitute a distinctive subset characterized by a fluid-filled sac arising from the greater omentum. We present the case of a three-year-old male who presented with severe abdominal pain localized to the right upper quadrant, progressively worsening over 24 hours. Physical examination revealed tenderness and a palpable mass. Laboratory investigations indicated mild leukocytosis. Contrast-enhanced computed tomography identified an omental cyst with torsion. Intraoperatively, the cyst arising from the greater omentum exhibited torsion, leading to ischemic changes. Surgical excision successfully corrected the torsion and removed the cyst. Omental torsion is a rare complication of omental cysts. Prompt recognition and surgical intervention are crucial, emphasizing the importance of considering diverse etiologies in acute pediatric abdominal pain.

## Introduction

Acute abdominal pain in pediatric patients presents a diagnostic challenge due to the diverse etiologies that range from benign to potentially life-threatening conditions. Omental cysts, though infrequent, represent a distinct category of abdominal pathology characterized by a fluid-filled sac originating from the greater omentum [1]. While these cysts are generally benign, their occurrence in children, particularly when accompanied by torsion of the greater omentum, is a rare clinical occurrence. There is limited literature on pediatric cases involving omental cysts with torsion [2-5]. Despite the usual lack of symptoms in the majority of omental cysts, torsion of the greater omentum can result in acute abdominal pain, emphasizing the need for timely recognition and intervention [3]. This case report details an instance of omental cyst torsion in a pediatric patient presenting with acute abdominal pain.

## Case presentation

A three-year-old male child presented to the Pediatric Emergency Department with a chief complaint of severe abdominal pain. The parents reported that the pain had progressively worsened over the past 24 hours and was localized to the right upper quadrant of the abdomen. There was no history of trauma or significant illness preceding the onset of symptoms.

The child had a normal birth history, characterized by an uneventful pregnancy, full-term delivery, and no notable complications during the perinatal period. Developmental milestones were age-appropriate, with the child achieving expected milestones such as rolling over, sitting, crawling, and walking at appropriate ages. There were no significant delays or concerns noted in the child's developmental progression.

Upon physical examination, the patient appeared distressed and was guarding his abdomen. Vital signs were within normal limits for age. Specifically, the heart rate was 110 beats per minute, the respiratory rate was 20 breaths per minute, the blood pressure was 90/60 mmHg, and the temperature was 37.0 degrees Celsius. The child's weight at the time of presentation was 15 kg. Abdominal examination revealed localized tenderness and a palpable mass in the right upper quadrant. The rest of the systemic examination was unremarkable. Given the acuity and intensity of the abdominal pain, coupled with the physical findings, an urgent work-up was initiated.

Laboratory investigations were notable for a mild leukocytosis (white blood cell count of 12,000/mm³) with a left shift. Serum electrolytes, liver function tests, and amylase levels were within normal ranges (Table 1). To further delineate the abdominal mass and assess for any underlying pathology, a contrast-enhanced computed tomography scan was performed.

**Table 1:** Laboratory results on presentation

Lab test	Result	Reference range
Hemoglobin	12.5 g/dL	11.5–14.5 g/dL
White blood cell count	12,000/mm³	4,000–11,000/mm³
Platelet count	300,000/mm³	150,000–450,000/mm³
Sodium	138 mmol/L	135–145 mmol/L
Potassium	4.2 mmol/L	3.5–5.0 mmol/L
Chloride	100 mmol/L	98–106 mmol/L
Alanine aminotransferase	25 U/L	7–56 U/L
Aspartate aminotransferase	30 U/L	10–40 U/L
Total bilirubin	0.5 mg/dL	0.3–1.2 mg/dL
Amylase	50 U/L	30–110 U/L

**Figure 1:**
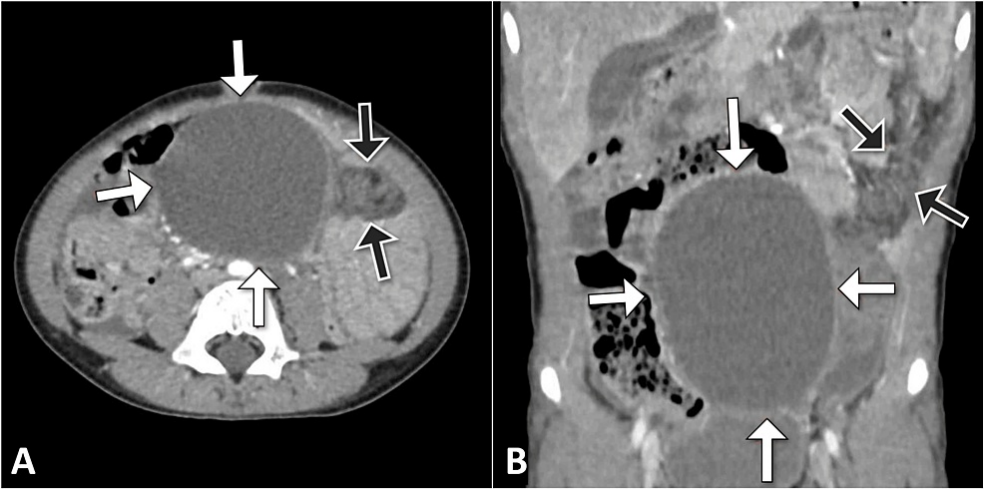
Axial (A) and coronal (B) CT images of the abdomen reveal a well-defined cystic lesion in the mid-abdomen (white arrow), accompanied by an adjacent area of fat density featuring distinctive streaks of whirling and concentric patterns (grey arrow). These radiological findings are indicative of an omental cyst with torsion CT: computed tomography

The computed tomography scan revealed a well-defined oval-shaped cystic lesion in the mid-abdomen with no internal septation, calcification, or soft tissue component. The cyst measured 8.2 x 4.7 x 3.4 cm on maximum dimensions. Notably, adjacent to the cystic mass, there was a fat density lesion that exhibited streaks of whirling and concentric patterns, which were associated with the omental cyst and torsion (Figure 1).

These radiological findings were highly indicative of an omental cyst with torsion, underscoring the vascular compromise associated with the torsion that contributed to the acute abdominal pain experienced by the patient. The absence of concerning features such as septation or calcification helped further establish the diagnosis. This imaging supported the decision for surgical exploration and subsequent excision of the omental cyst to alleviate the torsion and prevent potential complications.

**Figure 2:**
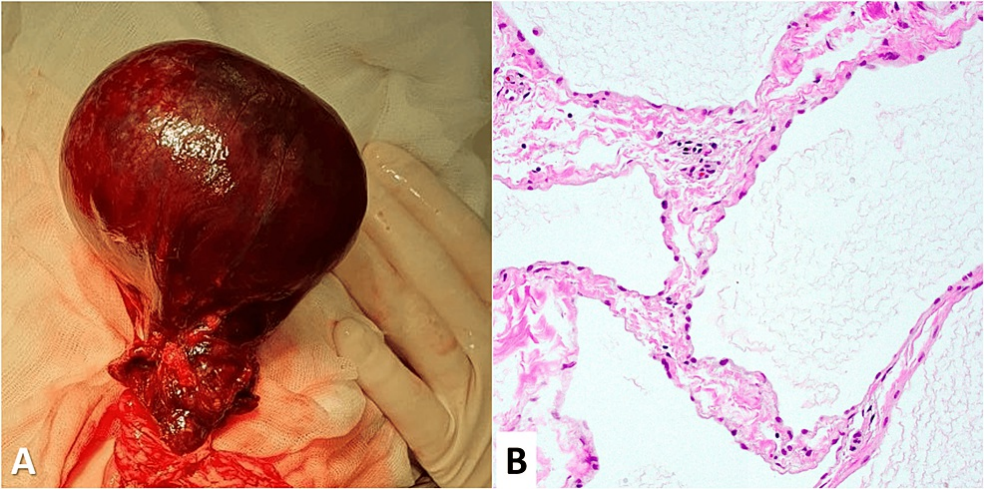
Intraoperative photography (A) captures the omental cyst, while histopathological examination (B) reveals cystic lesions characterized by flattened epithelium and occasional inflammatory infiltrates

Intraoperatively, a cystic mass arising from the greater omentum was identified and confirmed. Torsion of the omental pedicle was evident, leading to compromised blood supply and ischemic changes within the omental tissue. The cyst was carefully dissected, and the torsion was corrected. Postoperative histopathological examination confirmed the diagnosis of an omental cyst with evidence of hemorrhagic infarction (Figure 2).

The postoperative course was uneventful, with the child gradually recovering from the abdominal pain. Intravenous antibiotics were administered postoperatively to prevent any potential infection. The patient was closely monitored for signs of complications, and oral intake was reintroduced once bowel function returned to normal.

## Discussion

Omental cysts are cystic lesions arising from the omentum, a double-layered fold of the peritoneum that hangs down from the stomach and covers the abdominal organs [4]. While the prevalence rate is not explicitly discussed in this context, it is noteworthy that omental cysts are considered rare entities in the medical literature. The etiology and pathogenesis of omental cysts, though not definitively elucidated, involve multifaceted mechanisms. Proposed hypotheses include developmental anomalies, suggesting abnormal embryological processes contribute to cystic degeneration later in life, and trauma, either acute or repetitive, triggering cystic changes in the omentum. Inflammatory processes, such as infections or chronic irritation, have also been implicated. The diversity of histopathological subtypes, including lymphatic malformations, mesothelial cysts, and pseudocysts, adds complexity to understanding omental cyst formation [1,2].

The clinical presentation of omental cysts encompasses a spectrum of manifestations, with abdominal pain emerging as the predominant symptom. Patients commonly report localized dull or colicky pain, often associated with gradual or sudden onset. Abdominal distension may accompany cyst enlargement, resulting in a palpable mass upon examination. Gastrointestinal symptoms such as nausea, vomiting, and alterations in bowel habits can arise, attributed to mechanical pressure on the gastrointestinal tract [6,7]. Notably, incidental findings through imaging studies underscore the importance of considering omental cysts in asymptomatic cases [3]. Rarely, complications like torsion, bleeding, or rupture can lead to an acute abdomen.

Accurate diagnosis of omental cysts is imperative for guiding appropriate management strategies. Ultrasound serves as a valuable initial screening tool, offering real-time imaging and aiding in the identification of cystic structures within the omental region. In the evaluation of intra-abdominal cystic lesions, sonography plays a pivotal role as a non-invasive and readily available imaging tool. It provides real-time visualization, aiding in distinguishing fluid-filled structures from solid masses and offering insights into the characteristics of the cyst [2-5]. Computed tomography scans provide detailed cross-sectional images, facilitating precise localization, characterization, and assessment of the cyst's relationship with adjacent structures. Fine-needle aspiration may be utilized in certain cases to obtain fluid for cytological analysis, confirming the cystic nature and excluding other pathological entities [1,6].

In considering the differential diagnosis of intra-abdominal cystic lesions, it is imperative to explore a range of potential etiologies to ensure accurate and comprehensive clinical evaluation. Cysts within the abdominal cavity may arise from various structures, including the liver, pancreas, kidneys, and reproductive organs. Common differential considerations encompass cystic neoplasms, such as pancreatic cystic neoplasms or liver cystadenomas, as well as congenital cystic lesions like choledochal cysts or renal cysts. Additionally, infectious etiologies, such as abscesses or hydatid cysts, and inflammatory conditions like pseudocysts, should be considered [3-5].

The optimal management of omental cysts involves an individualized approach, considering factors such as the size and location of the cyst, associated symptoms, and the overall health status of the patient [3]. Surgical excision stands as the primary and most widely accepted intervention, particularly for symptomatic or large cysts. The surgical approach may vary from laparoscopic to open methods, depending on the complexity of the cyst and the surgeon's expertise [5]. However, a conservative approach, including observation and periodic imaging, may be considered for asymptomatic or small cysts, especially in cases where surgery poses a higher risk. Percutaneous drainage, guided by imaging, is another alternative, particularly in cysts with clear fluid content [3]. In our case, emergent surgery was essential as the patient had an omental cyst with omental torsion.

## Conclusions

This case report underscores the diagnostic and therapeutic challenges posed by omental cysts with torsion in pediatric patients, emphasizing the significance of prompt recognition and intervention. The rare prevalence of such cases underscores the importance of considering diverse etiologies in the assessment of acute abdominal pain in children. The detailed presentation of the patient's history, clinical examination, laboratory results, and imaging findings contributes to a comprehensive understanding of this uncommon clinical entity. The successful intraoperative correction of torsion and excision of the omental cyst, guided by careful preoperative assessment, exemplify the effective management of this condition.
